# Derivation and validation of a prediction model for primary and recurrent *Clostridioides difficile* infection among the hematopoietic cell transplantation population

**DOI:** 10.1017/ash.2026.10315

**Published:** 2026-03-25

**Authors:** Joseph O’Brien, Monica Nair, Betty Hamilton, Matthew A. Pappas, Michael B. Rothberg, Abhishek Deshpande

**Affiliations:** 1 Department of Emergency Medicine, Denver Health Medical Center: Denver Health Main Campus, Denver, USA; 2 Department of Internal Medicine, Massachusetts General Hospital, Boston, USA; 3 Department of Hematology and Medical Oncology, Cleveland Clinic Foundation: Cleveland Clinic, USA; 4 Center for Value-Based Care Research, Primary Care Institute, Cleveland Clinic Health System: Cleveland Clinic, USA; 5 Center for Value-Based Care Research, Primary Care Institute, Cleveland Clinic Main Campus Hospital: Cleveland Clinic. Cleveland, USA

## Abstract

**Objective:**

*Clostridioides difficile* infection (CDI) disproportionately affects hematopoietic cell transplantation (HCT) recipients. causing significant morbidity and mortality. This study aimed to develop and validate clinical prediction models for primary and recurrent CDI within one year post-transplant in this high-risk population.

**Methods:**

We conducted a retrospective cohort study of HCT recipients (2010–2023) at a single institution. The cohort was randomly split into derivation (70%) and internal validation (30%) sets. We compared logistic regression with backward elimination by Akaike information criterion (AIC), random forest, and LASSO regularization approaches. Candidate predictors included demographics, clinical variables, laboratory values, and medication exposures. Model discrimination was assessed using the C-statistic, and calibration by observed vs. predicted proportions across risk deciles.

**Results:**

Among 2,725 HCT recipients, 252 (9.3%) developed CDI within one year, and 22 (8.7% of primary CDI cases) developed recurrence. For primary CDI, the backward elimination model performed best, including five predictors: receipt of cephalosporins (OR 1.46; 95% CI, 1.02–2.11), sulfonamides (OR 1.78; 95% CI, 1.04–3.05), penicillins (OR 1.4; 95% CI, 0.96–2.02), autologous transplant (OR 0.39; 95% CI, 0.22–0.66), and insurance type (Medicare: OR 30.2; 95% CI, 16.9–53.6; Medicaid: OR 15.2; 95% CI 6.5–35.4). This model showed good discrimination (C-statistic: 0.81 in both derivation and validation cohorts) with adequate overall calibration. For recurrent CDI, elevated white blood cell count at primary diagnosis was the only independent predictor (OR 1.16; 95% CI, 1.06–1.27), with modest discrimination (C-statistic: 0.73 derivation, 0.70 validation).

**Conclusion:**

We derived and internally validated prediction models for CDI in HCT recipients which could facilitate targeted preventive interventions in this high-risk population.

## Introduction

*Clostridioides difficile* (*C. difficile*) infection (CDI) is the leading cause of healthcare-associated diarrhea in the United States and causes considerable morbidity and mortality.^1,2^ Hematopoietic cell transplantation (HCT) recipients are disproportionately affected by CDI and have worse clinical outcomes, including fulminant colitis and toxic megacolon.^3–11^ Previous studies have demonstrated that interventions such as environmental cleaning, enhanced hand hygiene protocols, and antibiotic stewardship can reduce CDI transmission and incidence.^12,13^

Several prediction models have been developed for CDI in the general hospitalized population, with varying levels of performance.^14–16^ However, these models have limited utility for HCT patients due to the unique risk factors and higher baseline incidences ranging from 12.5 to 30% within in this population.^16–20^ Previous studies have identified risk factors for primary and recurrent CDI among HCT patients, but these were primarily descriptive and did not develop or validate prediction models.^6,21–24^ To our knowledge, no published studies have described comprehensive prediction models specifically for CDI among HCT patients.

The objective of this study was to derive and internally validate clinical prediction models for both primary and recurrent CDI among HCT recipients. While previous studies have described risk factors in this cohort, they have primarily remained descriptive and lacked validated predictive tools. This study focuses on the methodologic development and internal validation of such models, representing an early step toward future risk stratification. Importantly, these tools are intended as a framework for identifying high-risk subsets for future research and are not proposed for immediate clinical implementation.

## Methods

### Study population

We assembled a cohort of HCT recipients from the Unified Transplant Database, a prospectively maintained database of autologous and allogenic HCT recipients at the Cleveland Clinic, Cleveland, Ohio, USA. The study was approved by the Cleveland Clinic Institutional Review Board. We included adults (≥18 yr) with hematological malignancy and/or bone marrow failure syndromes who underwent their first HCT between January 1, 2010, and March 31, 2023. Patients with a history of CDI diagnosis within 8 weeks prior to HCT were excluded.

We identified three outcome categories based on CDI diagnoses after engraftment: (1) patients without CDI, (2) patients with primary CDI only, and (3) patients with primary and recurrent CDI (Supplemental Figure 1). All outcomes were assessed through 1 year following transplant. Primary CDI was defined according to Infectious Disease Society of America/Society for Healthcare Epidemiology of America (IDSA/SHEA) guidelines as new-onset diarrhea (≥3 unformed stools within 24 h) and a positive stool test for *C. difficile*.^25^ Recurrent CDI was defined as diarrhea and a positive CDI stool test between 2 and 8 weeks after completion of treatment for primary CDI, consistent with current clinical guidelines.^25^

To evaluate the possible influence of changes in CDI diagnostic methodology on incidence rates, we categorized patient encounters into three testing eras based on institutional laboratory protocols: EIA-only (January 1, 2010—October 31, 2010), PCR-only (November 1, 2010—May 31, 2018), and PCR plus EIA confirmation (June 1, 2018—March 31, 2023). We performed logistic regression analyses with CDI status as the outcome and testing era as the primary predictor to evaluate differences in CDI detection across these periods.

### Candidate predictor variables

Potential predictors for primary and recurrent CDI were selected through comprehensive literature review, focusing on predictors that would be available at the time of transplant (for prediction of primary CDI) or at the time of primary CDI diagnosis (for prediction of recurrent CDI). For prediction of primary CDI, candidate predictors included demographics (age, sex, race, body mass index (BMI), and insurance type categorized as private, Medicare, Medicaid, or other), clinical characteristics (hematopoietic cell transplantation-specific comorbidity index (HCT-CI), primary disease, and presence of specific comorbidities including inflammatory bowel disease, chronic kidney disease, and congestive heart failure), and transplant factors (donor type, stem cell source, and conditioning regimen intensity). We also collected healthcare utilization data (total hospital visits in the year preceding HCT admission, including number of inpatient days and outpatient visits), medication exposure (receipt of antibiotics including cephalosporins, penicillins, quinolones, clindamycin, macrolides, sulfonamides, and tetracyclines, as well as gastric acid suppressors, immunosuppressive agents, antidiarrheals, opioids, and total parenteral nutrition within 90 d prior to transplant), and laboratory measures (white blood cell count, serum creatinine, blood urea nitrogen, and serum albumin). For prediction of recurrent CDI, we collected the same variables but measured at the time of primary CDI diagnosis. For WBC count and serum creatinine, we recorded both the value on the day of transplant (or primary CDI diagnosis) and the highest value during the admission.

Continuous variables (eg, white blood cell count, serum creatinine, serum albumin) were assessed for nonlinearity and retained in their native scale, as transformation did not improve model fit. Categorical predictors (eg, graft type, stem cell source, hematologic malignancy subtype) were converted into indicator variables. Binary predictors represented medication exposures and clinical factors that were either present or absent within a defined period (eg, receipt of cephalosporins, penicillins, sulfonamides, gastric acid suppression, or immunosuppressive therapy). Missingness for all included variables was <5%; therefore, a complete-case approach was used.

Medication exposures were measured within 90 d prior to transplant (or prior to the index CDI diagnosis for the recurrence model) to capture the clinically relevant period of antibiotic-associated gut microbiome disruption.^6,21^

We selected a one-year follow-up period to capture the period of highest CDI risk following HCT, including early postengraftment and delayed infectious complications. Limiting the observation window to one year minimizes potential confounding associated with long-term survivorship and evolving exposures.

### Statistical methods

Derivation and internal validation cohorts: We randomly divided the data set with 70% serving as the derivation cohort and the remaining 30% used for the internal validation cohort (Supplemental Figure 2). We repeated this approach in the population with primary CDI to create a model to predict CDI recurrence.

In the derivation cohort for primary CDI prediction, all candidate predictor variables were included as potential covariates in a multivariable logistic regression model using CDI within 1 year posttransplant as the primary outcome. To obtain a parsimonious model, we used three different approaches. First, backward elimination by Akaike information criterion (AIC) was employed. Second, we applied least absolute shrinkage and selection operator (LASSO) regularization with 10-fold cross-validation to select the optimal penalty parameter (lambda) that minimized deviance. Third, we developed a random forest model with 500 trees, a variable selection threshold of the square root of the total predictor count per split, a minimum terminal node size of 1, and an unrestricted number of maximum nodes per tree.^26,27^ Variables were ranked by importance using Gini impurity.

The final model from each approach was further simplified by removing variables that contributed little to discrimination, as assessed by the change in C-statistic, based on clinical and statistical judgment. We compared model discrimination using the C-statistic (area under the receiver operating characteristic curve). We assessed model calibration by grouped predicted risk graphically by comparing observed and predicted proportions of patients who developed CDI within increasing deciles of predicted probability. For interpretability, we evaluated model performance at a 10% predicted-risk threshold, corresponding approximately to the observed 9.3% incidence of CDI in the cohort, to identify patients at above-average risk

This approach was repeated to generate a model for recurrent CDI. The first model output was the probability that a patient would develop primary CDI, and the second model output was the probability a patient would develop recurrent CDI. All analyses were performed using R statistical software (version 4.1.0), with the *glmnet* package for regression models and *randomForest* package for random forest models.

## Results

### Characteristics of the study population

Among 2,768 patients who underwent HCT during the study period, 2,725 met inclusion and exclusion criteria. Of these, 252 (9.3%) developed CDI within 1 year from transplant (Supplemental Figure 1). Among patients with primary CDI, 22 (8.7% of primary cases, 0.8% of the entire study population) developed recurrent CDI within 1 year. The incidence of CDI varied by transplant type, with 17.8% (181 of 1,016 patients) in allogenic HCT recipients compared to 4.1% (71 of 1,709 patients) in autologous recipients.

Table 1 presents the demographic and clinical characteristics of the study population stratified by CDI status. Patients with primary CDI had higher rates of specific comorbidities, including inflammatory bowel disease (6.7% vs 4.8%) and congestive heart failure (11.5% vs 8.0%). Another difference between the groups was insurance status. Patients who developed CDI were significantly less likely to have private insurance compared with patients without CDI (64.7% vs 97.5%).

**Table 1.** Demographic and clinical characteristics of HCT recipients stratified by CDI status

Characteristic	No CDI (*n* = 2,473)	Primary CDI (*n* = 252)	Recovered from CDI (*n* = 230)	Recurrent CDI (*n* = 22)
**Demographics**				
Age, years, mean (SD)	65.7 (15.6)	65.9 (15.5)	66.0 (15.7)	66.5 (14.8)
Sex, male, *n* (%)	1,425 (57.6)	152 (60.3)	140 (60.9)	12 (54.5)
BMI, kg/m^2^, mean (SD)	29.8 (6.4)	29.2 (5.8)	29.3 (5.9)	22.2 (5.0)
Race, *n* (%)				
White	2,141 (86.6)	220 (87.3)	230 (86.5)	21 (95.5)
Black	233 (9.4)	20 (7.9)	19 (8.3)	1 (4.6)
Other	99 (4.0)	12 (4.8)	12 (5.2)	0 (0.0)
Insurance, *n* (%)				
Private	2,412 (97.5)	163 (64.7)	150 (65.2)	13 (59.1)
Medicare	42 (1.7)	66 (26.2)	58 (25.2)	8 (36.4)
Medicaid	19 (0.8)	23 (9.1)	22 (9.6)	1 (4.6)
**Clinical characteristics**				
HCT-CI, *n* (%)				
0–1	893 (36.1)	70 (27.8)	60 (26.1)	10 (45.4)
2	422 (17.1)	45 (17.9)	40 (17.4)	5 (22.7)
≥3	1,158 (46.8)	137 (54.4)	130 (56.5)	7 (31.8)
Comorbidities, *n* (%)				
Inflammatory bowel disease	119 (4.8)	17 (6.7)	12 (5.7)	4 (18.2)
Chronic kidney disease	120 (4.9)	8 (3.2)	6 (2.6)	2 (9.1)
Congestive heart failure	199 (8.0)	29 (11.5)	26 (11.3)	3 (13.6)
Total hospital visits within 1 year, median (IQR)	18 (19)	19.5 (17)	19 (17)	22 (19)
Prior chemotherapy regimens, *n* (%)				
None	174 (7.0)	4 (1.6)	4 (1.7)	0 (0.0)
1–3 regimens	2,110 (85.3)	225 (89)	22 (9.6)	21 (95.4)
>3 regimens	189 (7.6)	23 (9.1)	204 (88.7)	1 (4.5)
**Hematological malignancy**				
AML	337 (13.6)	85 (33.7)	79 (34.3)	6 (27.3)
MDS	168 (6.8)	41 (16.3)	38 (16.5)	3 (13.6)
ALL	84 (3.4)	20 (7.9)	18 (7.8)	2 (9.1)
Chronic myeloproliferative neoplasm	248 (10.0)	23 (9.1)	19 (8.3)	4 (18.1)
Multiple myeloma	892 (36.1)	41 (16.3)	38 (16.5)	3 (13.6)
NHL	392 (15.9)	19 (7.5)	16 (7.0)	3 (13.6)
Other	352 (14.2)	23 (9.1)	22 (9.6)	1 (4.5)
**Transplant factors**				
Stem cell source, *n* (%)				
Bone marrow	289 (11.7)	62 (24.6)	60 (26.1)	2 (9.1)
Peripheral blood	2,058 (83.2)	167 (66.3)	149 (64.8)	18 (81.8)
Cord blood	126 (5.1)	23 (9.1)	21 (9.1)	2 (9.1)
Graft type, *n* (%)				
Autologous	1,638 (66.2)	71 (28.2)	65 (28.3)	6 (27.3)
Allogenic	835 (33.8)	181 (71.8)	165 (71.7)	16 (72.7)
**Medication exposure**				
Total number of antibiotic classes, *n* (%)				
0–1 classes	1,207 (48.8)	80 (31.7)	29 (12.6)	3 (13.6)
2–3 classes	1,159 (46.8)	159 (63.0)	149 (64.8)	12 (54.5)
>3 classes	107 (4.3)	13 (5.2)	52 (22.6)	7 (31.8)
Antibiotics within 90 days, *n* (%)				
Cephalosporins	1,426 (57.7)	145 (57.5)	121 (52.6)	10 (45.4)
Penicillins	797 (32.2)	138 (54.8)	171 (74.3)	16 (72.3)
Quinolones	2,034 (82.2)	165 (65.5)	133 (57.8)	14 (63.6)
Clindamycin	60 (2.4)	12 (4.8)	8 (3.5)	1 (4.5)
Macrolides	373 (15.1)	27 (10.7)	32 (13.9)	8 (36.4)
Sulfonamides	921 (37.2)	178 (70.6)	155 (67.4)	12 (54.5)
Tetracyclines	76 (3.1)	8 (3.2)	5 (2.2)	1 (4.5)
Acid suppression, *n* (%)	2,451 (99.1)	250 (99.2)	214 (93.0)	19 (86.4)
Immunosuppressive agents, *n* (%)	2,463 (99.6)	248 (98.4)	228 (99.1)	20 (90.9)
Antidiarrheal agents, *n* (%)	401 (16.2)	66 (26.2)	94 (40.9)	9 (40.9)
Opioids, *n* (%)	2,471 (99.9)	252 (100)	220 (95.7)	20 (90.9)
**Laboratory values**				
WBC (x10^9^/L), median (IQR)	1.16 (2.0)	0.82 (1.8)	2.45 (5.74)	4.75 (3.19)
Max WBC during admission (x10^9^/L), median (IQR)	5.28 (4.0)	4.96 (4.2)	4.4 (7.23)	5.8 (4.28)
Max Creatinine during admission (mg/dL), median (IQR)	0.86 (0.37)	0.89 (0.39)	0.93 (0.42)	1.03 (0.69)
Bilirubin (mg/dL), median (IQR)	0.5 (0.3)	0.45 (0.4)	0.5 (0.4)	0.6 (0.6)
Glucose (mg/dL), median (IQR)	98 (22)	98.5 (22)	105 (33)	109 (33.5)
AST (units/L), median (IQR)	18 (10)	18 (11.3)	20 (15)	24 (18.2)
Albumin (g/L), median (IQR)	3.5 (0.5)	3.5 (0.4)	3.4 (0.8)	3.5 (0.8)
BUN (mg/dL), median (IQR)	12 (6)	13 (7)	14 (12)	16 (10)

Numerical variables are displayed as mean ± standard deviation or median (IQR), as appropriate. Categorical variables are displayed as number of patients, followed by percentage. Medication data for patients with and without primary CDI is within 90 days from transplant; medication data for patients with recurrent CDI and those who recovered is within 90 days prior to the primary CDI diagnosis. Laboratory data for patients with or without primary CDI is from the day of transplant; laboratory data for patients with recurrent CDI and those who recovered is from the date of primary CDI diagnosis.

ALL, acute lymphoblastic leukemia. AML, acute myelogenous leukemia. BMI, body mass index. CI, confidence interval. CML, chronic myelogenous leukemia. CHF, chronic heart failure. CKD, chronic kidney disease. HCT-CI, Hematopoietic Cell Transplantation (HCT)-specific Comorbidity Index. IBD, inflammatory bowel disease. IQR, interquartile range. MDS, myelodysplastic syndrome. NHL, Non-Hodgkin lymphoma. SD, standard deviation. WBC, white blood cell. BUN, blood urea nitrogen.

Regarding medication exposures, patients who developed primary CDI were more likely to have received penicillins (54.8% vs 32.2%) or sulfonamide antibiotics (70.6% vs 37.2%) within 90 days prior to transplant.

Among the 252 patients who developed primary CDI, 22 (8.7%) developed a first recurrence, 12 (4.8% of index cases; 54.5% of first recurrence cases) developed a second recurrence, and 8 (3.2% of index cases; 75% of second recurrence cases) developed a third recurrence. Patients with recurrent CDI had higher rates of inflammatory bowel disease (18.2% vs 5.7%) and chronic kidney disease (9.1% vs 2.6%) compared to those who recovered.

Laboratory findings showed that patients who developed recurrent CDI had more pronounced leukocytosis on the day of primary CDI diagnosis (median 4,750 vs 2,450 cells/mm^3^) and during the admission for primary CDI treatment (median 5,800 vs 4,400 cells/mm^3^).

The incidence of CDI did not significantly differ across the defined diagnostic eras. In logistic regression models, the odds of CDI diagnosis during the PCR-only era were not significantly different compared to the EIA-only era (OR .70, 95% CI .21–2.17, *P* = .54). Likewise, the PCR plus EIA era demonstrated no significant difference in CDI odds relative to the EIA era (OR .82, 95% CI .24–2.52, *P* = .73).

### Predictors of primary CDI in the derivation cohort

Table 2 presents the results from our models for predicting primary CDI. The logistic regression model with backward elimination by AIC performed best and included five predictors: receipt of cephalosporins, penicillins, sulfonamides, transplant type, and insurance status. Receipt of cephalosporins (OR 1.46; 95% CI, 1.02–2.11), sulfonamides (OR 1.78; 95% CI, 1.04–3.05), and penicillins (OR 1.40; 95% CI, 0.96–2.02) within 90 days prior to transplant were associated with an increased risk of developing primary CDI. Autologous transplant (compared to allogenic) was associated with decreased odds, with an OR of 0.39 (95% CI, 0.22–0.66). With private insurance as the reference, Medicare (OR 30.2; 95% CI, 16.9–53.6) and Medicaid (OR15.2; 95% CI, 6.5–35.4) were associated with increased odds of developing primary CDI. Results from alternative modeling strategies (random forest and LASSO regularization) are presented in Supplementary Tables 2 and 3. These models showed lower discrimination compared to the backward elimination approach.

**Table 2.** Final prediction models for primary and recurrent CDI

Predictor	Odds ratio (95% CI)	*P*-value
**Primary CDI model**		
Receipt of cephalosporins	1.46 (1.02–2.11)	.04
Receipt of sulfonamides	1.78 (1.04–3.05)	.03
Receipt of penicillins	1.40 (0.96–2.02)	.08
Autologous transplant	0.39 (0.22–0.66)	<.001
Insurance (ref: Private)		
Medicare	30.2 (16.9–53.6)	<.001
Medicaid	15.2 (6.5–35.4)	<.001
**Recurrent CDI model**		
WBC count on day of primary CDI diagnosis (per 1,000 cells/mm^3^ increase)	1.16 (1.06–1.27)	.001

CI, confidence interval. WBC, white blood cell.

Given the unexpectedly strong association with insurance status, we conducted a post hoc analysis replacing insurance with age over 65 years, chronic kidney disease, and area deprivation index. This alternative model demonstrated significantly worse performance (C-statistic = 0.70; DeLong test, *P* = 0.02) compared to the original model including insurance status, suggesting that insurance captures additional predictors beyond age, kidney disease, and socioeconomic status.

### Primary CDI prediction model performance

We compared three modeling strategies—backward-elimination logistic regression, LASSO regression, and random forest—for predicting primary CDI within one year posttransplant. The backward-elimination logistic model demonstrated the best overall discrimination, with an AUC of 0.81 (95% CI, 0.75–0.86) in both the derivation and internal-validation cohorts. The LASSO model showed lower discrimination (AUC = 0.68, 95% CI, 0.63–0.73), and the random forest model performed modestly (AUC = 0.70, 95% CI, 0.65–0.75). These findings indicate that the backward-elimination logistic model provided the strongest and most stable predictive performance among the approaches tested and was therefore selected as the final primary CDI model.

**Figure 1.**
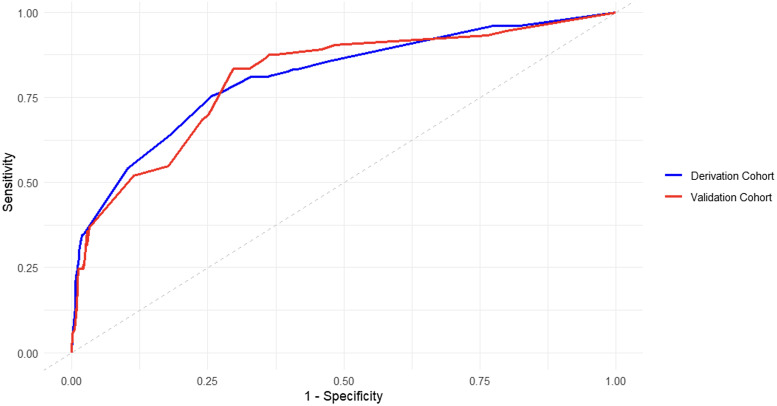
Receiver operating characteristic (ROC) curves for derivation and validation cohorts of primary CDI model.

Figure 1 displays the receiver operating characteristic curves for our primary CDI model in the derivation and validation cohorts. The model demonstrated good discrimination with a C-statistic of 0.81 in both the derivation and validation cohorts.

**Figure 2.**
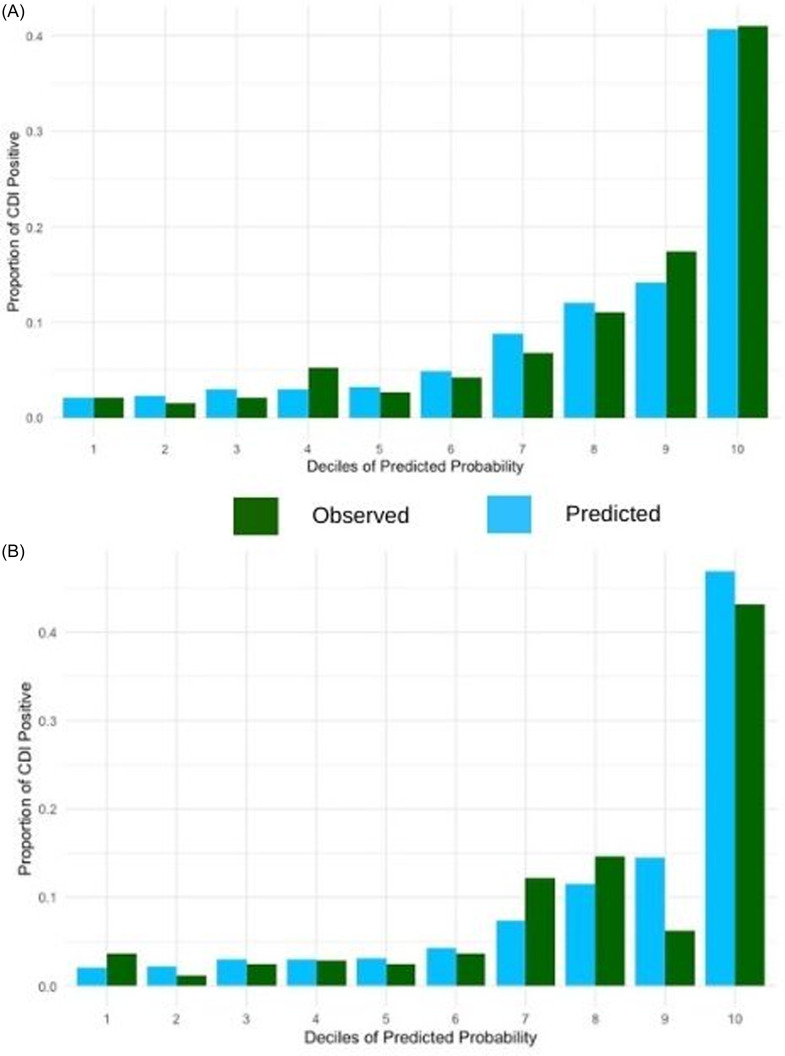
Calibration plot for the primary CDI model in the (A) derivation and (B) internal validation sets.

Figure 2 shows the calibration plots comparing observed and predicted proportions of patients who developed CDI within 1 year from transplant by deciles of predicted risk. The model demonstrated reasonable overall calibration in the validation set (Hosmer-Lemeshow goodness-of-fit, *P* > .2; Brier score 0.067). However, visual inspection revealed that the model underestimated risk in lower deciles (deciles 1–3) and overestimated risk in higher deciles (8 and 9). The observed proportions of patients who developed CDI were generally consistent with the predicted probabilities in the mid-range (deciles 4–7). At a 10% predicted-risk threshold, the model achieved sensitivity of 0.69, specificity of 0.76, positive predictive value (PPV) of 0.22, and negative predictive value (NPV) of 0.96 in the validation cohort, indicating good separation of high-and low-risk patients without performance inflation from the majority non-CDI class.

### Recurrent CDI prediction model

For predicting recurrent CDI, LASSO regression produced the model with the highest discrimination. This model included only one significant predictor: WBC count on the day of primary CDI diagnosis with a higher count associated with increased odds of developing recurrent CDI (OR 1.16; 95% CI, 1.06–1.27). Results from alternative modeling strategies (random forest and backward elimination) are presented in Supplementary Tables 4 and 5.

### Recurrent CDI prediction model performance

**Figure 3.**
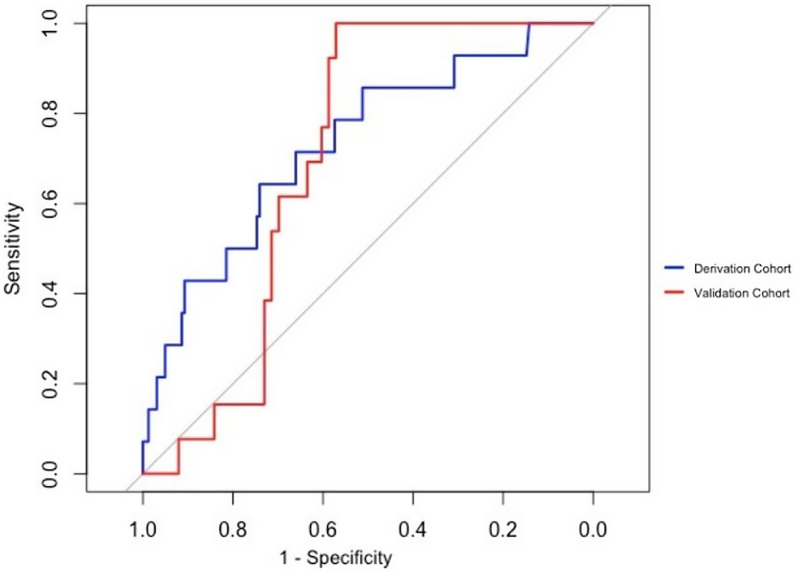
Receiver operating characteristic (ROC) curves for derivation and internal validation cohorts of recurrent CDI model.

**Figure 4.**
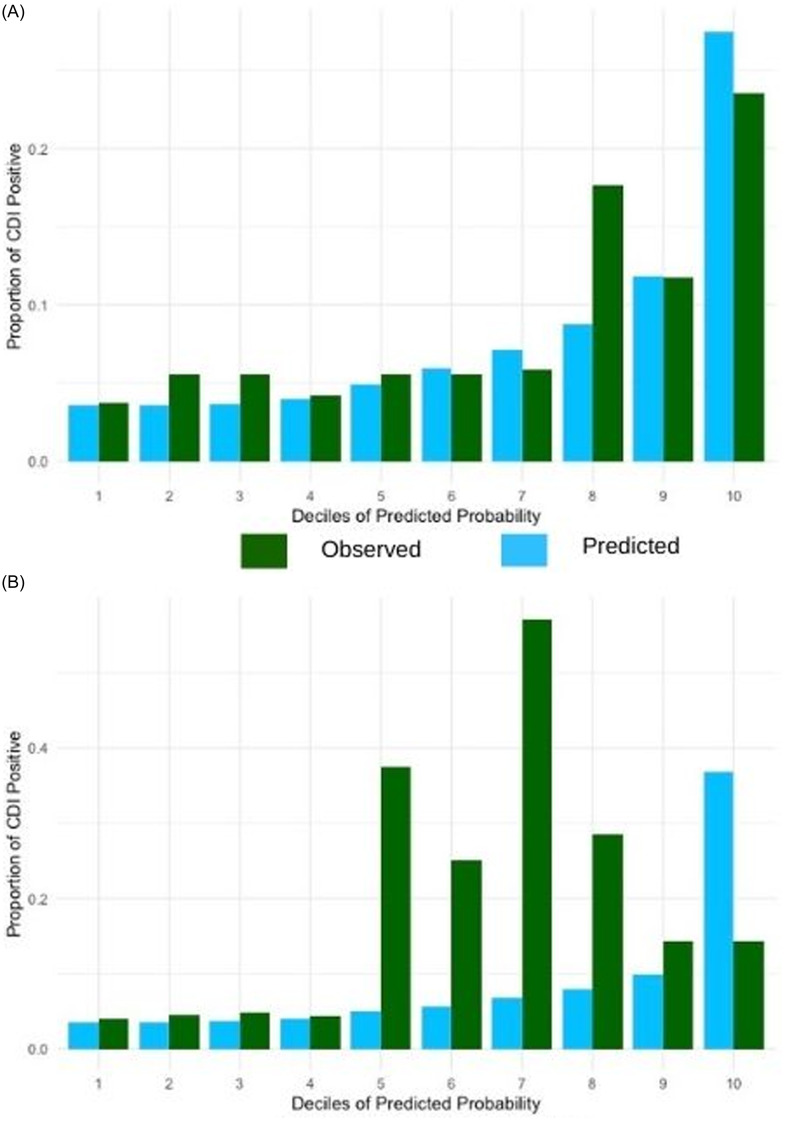
Calibration plot for the recurrent CDI model in the (A) derivation and (B) internal validation sets.

Figure 3 shows the receiver operating characteristic curves for our recurrent CDI model. The model had a C-statistic of 0.73 in the derivation cohort and 0.70 in the validation cohort, indicating moderate discrimination. Figure 4 shows the calibration plots comparing observed and predicted proportions of patients who developed recurrent CDI within 1 year after primary CDI by deciles of predicted risk. Calibration assessment for the model showed adequate overall fit (Hosmer-Lemeshow goodness-of-fit, *P* > .3; Brier score 0.075), though the small number of recurrent events (*n* = 22) limited our ability to perform detailed calibration analyses across risk deciles.

## Discussion

In this large retrospective cohort study, we derived and internally validated prediction models for primary and recurrent CDI among the HCT population. Our primary CDI model identified key predictors, including receipt of cephalosporins and sulfonamides, insurance type, and transplant type, achieving good discrimination with a C-statistic of 0.81. We also developed a clinical prediction tool for recurrent CDI based on WBC count at primary CDI diagnosis, which demonstrated moderate discrimination with a C-statistic of 0.70–0.73.

We evaluated multiple modeling approaches to balance predictive performance with clinical interpretability. Traditional logistic regression with backward elimination offers transparency, which is important for the practical application of infection prevention and antimicrobial stewardship programs. LASSO regression provides regularization to reduce overfitting in high-dimensional clinical data, while random forest methods allow for modeling of potential nonlinear relationships. In our cohort, the backward-elimination model showed the most stable performance suggesting that parsimonious models may be better suited for clinical settings characterized by relatively low event rates.

We identified cephalosporin use as a significant predictor for primary CDI among HCT recipients which is consistent with prior research.^28,29^ However, our finding that sulfonamide use was associated with increased CDI risk contrasts with a previous study by Lavallée et al., which reported sulfonamides as potentially protective in allogenic HCT patients.^21^ Notably, numerous studies in the general population have identified sulfonamides as a risk factor for both hospital-acquired and community-acquired CDI.^30–35^ The protective effect of autologous transplant (OR 0.39) is consistent with previous studies showing lower CDI incidence in autologous compared to allogenic HCT recipients.^6,7,21,36^

We observed a strong association between public insurance (Medicare/Medicaid) and risk of CDI. While one previous study found public insurance predictive of primary CDI among general hospitalized patients,^17^ the magnitude of association in our study was substantial. In post hoc analyses, replacing insurance status with age ≥65 years, chronic kidney disease, and area deprivation index reduced model performance. This suggests that insurance status captures additional risk factors beyond these measured variables. Medicare beneficiaries include a heterogeneous population, including younger patients with end-stage renal disease or disabilities, who may possess distinct comorbidity profiles. Therefore, insurance status likely functions as a proxy for unmeasured social determinants of health, geographic proximity to the transplant center, or disparities in healthcare access, rather than a direct causal factor. Insurance type may also reflect variation in pretransplant care, antibiotic exposure, and healthcare-seeking behaviors, all of which may influence CDI risk. These findings should therefore be interpreted cautiously, and identification of more granular social and healthcare-related contributors to CDI risk in transplant recipients remains an important area for future investigation.

For recurrent CDI, we identified elevated WBC count at primary CDI diagnosis as the only significant predictor, consistent with studies in the general hospitalized population.^37,38^ To our knowledge, this is the first study identifying severity of leukocytosis as a predictor for recurrent CDI among HCT recipients. However, given the low number of recurrent CDI cases, use of this model should be interpreted with caution.

Our primary CDI prediction model showed discrimination comparable to or better than models developed for the general hospitalized population.^14,15,17,18^ While overall calibration was adequate, the model tended to underestimate risk in lower-risk groups and overestimate risk in higher-risk groups. The observed sensitivity of 69% indicates that this tool represents an initial proof-of-concept and that additional refinement, potential incorporation of novel predictors, and external validation will be required to further improve performance characteristics. Ultimately, such models may support IDSA/SHEA-recommended preventive strategies by identifying high-risk patients for targeted antimicrobial stewardship and infection prevention measures. The model may also help identify patient subsets for enhanced surveillance or early intervention strategies but should not be used to guide treatment decisions without external validation.

Our recurrent CDI model showed modest discrimination, similar to existing models for the general population.^16,19,20,39^ The lower discrimination compared to our primary CDI model suggests that important predictors of recurrence such as subsequent antibiotic exposures and *C. difficile* ribotype may not be captured in current clinical data. Despite its limitations, the model could help identify patients who might benefit from extended CDI treatment courses or novel microbiome-based therapies or fecal microbiota transplantation to prevent recurrence.

Our clinical prediction tools have the potential to stratify patients into distinct CDI risk categories. This risk stratification can be used to guide targeted preventive interventions. For high-risk patients, a bundled approach could include enhanced infection control measures, antibiotic stewardship focused on high-risk antibiotics such as cephalosporins and sulfonamides, and prophylactic strategies such as oral vancomycin prophylaxis. Importantly, this prediction model should be viewed as a tool in development, rather than a platform ready for immediate clinical implementation. While the primary CDI model demonstrated good discrimination, further refinement and external validation are necessary before such models can reliably be incorporated into routine infection prevention practice or used to guide antimicrobial stewardship interventions.

Our study has several limitations. First, it was conducted at a single institution, with most patients identifying as White and having private insurance, which may limit the generalizability of the findings. However, this remains one of the largest, well-characterized cohorts of HCT recipients, spanning 13 years. Second, the strong association observed with insurance status requires further investigation to identify underlying causal factors. Third, changes in institutional CDI diagnostic testing criteria over time may have influenced our findings. Furthermore, we were unable to account for potential differences in CDI testing frequency across patient subgroups (eg, those with inflammatory bowel disease), which may have introduced detection bias, particularly during the PCR-only testing era when colonization could have been misclassified as infection. Although we excluded patients with CDI within 8 weeks prior to HCT, earlier CDI history may not have been captured and could confer residual risk, potentially leading to misclassification. Variation in CDI treatment strategies, including antimicrobial selection, duration of therapy, and use of adjunctive treatments, may influence recurrence risk and were not fully accounted for in this analysis. This heterogeneity may partially explain the modest discrimination of the recurrent CDI model and suggests that future studies should incorporate granular treatment data. Finally, our recurrent CDI model was limited by the small number of recurrent events (*n* = 22), which restricted our ability to identify multiple predictors and perform detailed calibration analyses.

## Supporting information

10.1017/ash.2026.10315.sm001 O’Brien et al. supplementary material O’Brien et al. supplementary material

## Supplementary material

The supplementary material for this article can be found at https://doi.org/10.1017/ash.2026.10315.
