# Supplementary material for: Derivation and validation of a prediction model for primary and recurrent Clostridioides difficile infection among the hematopoietic cell transplantation population
Source: Antimicrob Steward Healthc Epidemiol. 2026 Mar 25;6(1):e63. doi: 10.1017/ash.2026.10315 (PMC13104584; doi:10.1017/ash.2026.10315)
Supplement: O’Brien et al. supplementary material [file S2732494X26103155sup001.docx]

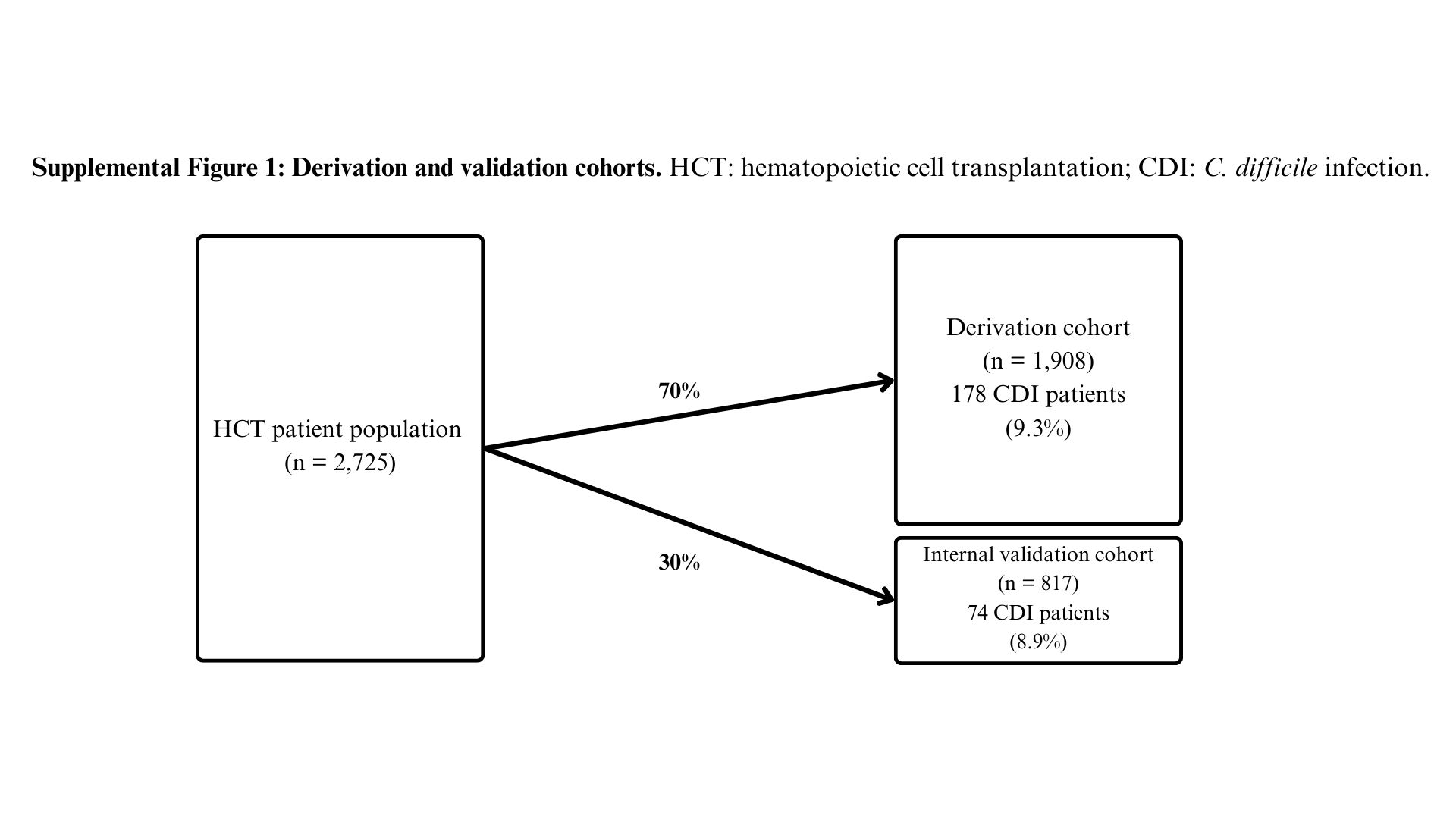


**Supplemental Figure 2**: **Study population**.
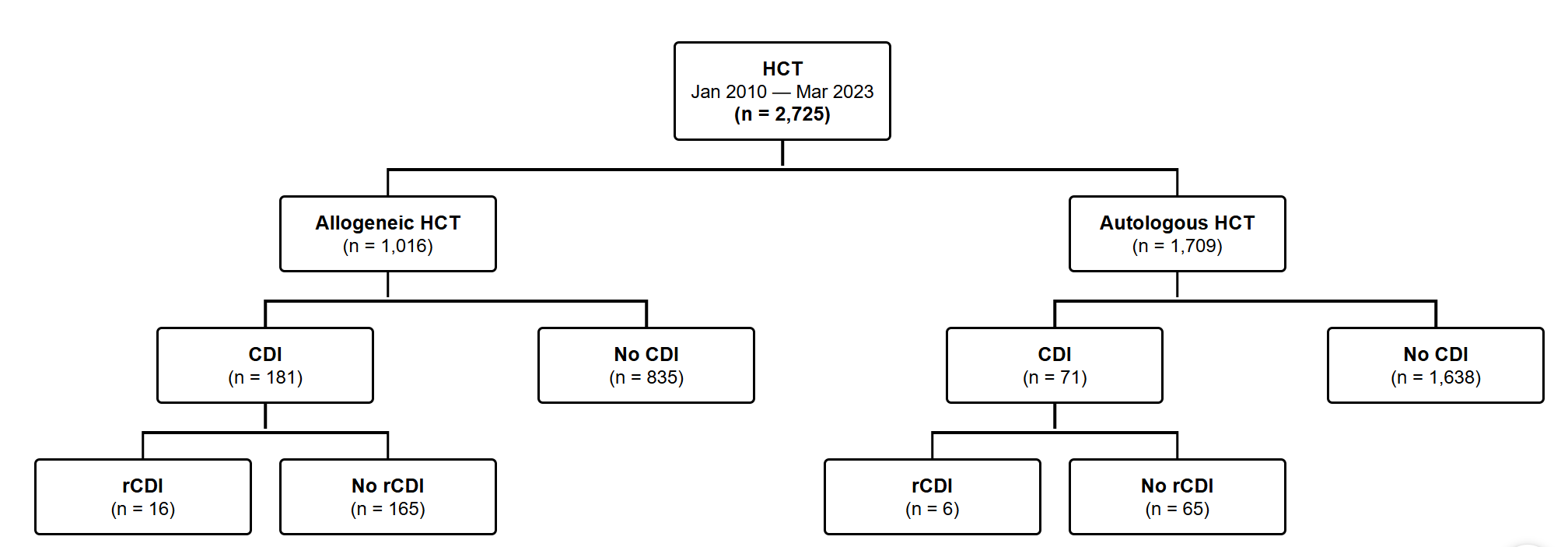


HCT: hematopoietic cell transplantation; CDI: *C. difficile* infection, rCDI: recurrent *C. difficle* infection

**Supplemental Table 1: Medication definitions.**

| **Medication Class** | **Medications** |
| --- | --- |
| Penicillins | Penicillin G, penicillin V, ampicillin, oxacillin, piperacillin, dicloxacillin, nafcillin, ampicillin, tazobactam, sulbactam, clavulanate |
| Cephalosporins | Ceftriaxone, cefixime, cefadroxil, cefpodoxime, ceftibuten, cefazolin, cefotaxime, cefdinir, ceftazidime, cephalexin, cefditoren, cefuroxime, cefepime, cefaclor |
| Quinolones | Levofloxacin, ciprofloxacin, moxifloxacin, Gemifloxacin, nalidixic acid, norfloxacin, ofloxacin, delafloxacin, cinoxacin, gatifloxacin, sparfloxacin, trovafloxacin |
| Clindamycin | — |
| Macrolides | Azithromycin, clarithromycin, fidaxomicin, erythromycin, telithromycin |
| Sulfonamides | Sulfamethoxazole/trimethoprim, sulfisoxazole |
| Tetracyclines | Tetracycline, doxycycline, minocycline, oxytetracycline, eravacycline, omadacycline |
| Acid suppression | Omeprazole, esomeprazole, lansoprazole, pantoprazole, dexlansoprazole, rabeprazole, vonoprazan, ranitidine, famotidine, nizatidine, cimetidine |
| Immunosuppressive agents | Cyclosporine, tacrolimus, azathioprine, prednisone, mycophenolate mofetil, sirolimus, everolimus, alemtuzumab |
| Opioids | Oxycodone, hydromorphone, methadone, tramadol, hydrocodone, naloxone, codeine, morphine, meperidine, buprenorphine, butorphanol, fentanyl, hydrocodone, oxymorphone, tapentadol |

**Supplemental Table 2: Associations between patient characteristics and primary CDI using Lasso regression in the derivation cohort.** C-statistic = 0.68 in the internal validation cohort

| Characteristic | Adjusted OR | 95% CI |
| --- | --- | --- |
| WBC | 1.0 | 0.99—1.01 |
| Penicillins | 1.34 | 0.94—1.91 |
| Sulfonamides | 1.35 | 0.84—2.20 |
| Antidiarrheals | 1.43 | 0.97—2.11 |
| Immunosuppression | 0.52 | 0.10—2.71 |
| Graft Type |  |  |
| Allogenic | Ref | Ref |
| Autologous | 0.48 | 0.24—0.97 |
| Stem cell source |  |  |
| Bone marrow | Ref | Ref |
| Peripheral blood | 0.90 | 0.59—1.38 |
| Cord blood | 1.40 | 0.75—2.64 |
| Hematological Malignancy |  |  |
| ALL—no.(%) | Ref | Ref |
| AML—no.(%) | 1.14 | 0.60—2.14 |
| MDS—no.(%) | 1.24 | 0.60—2.56 |
| Chronic myeloproliferative neoplasm—no.(%) | 0.64 | 0.29—1.42 |
| Other—no.(%) | 0.55 | 0.24—1.28 |
| Multiple myeloma—no.(%) | 0.72 | 0.30—1.76 |
| NHL—no.(%) | 0.57 | 0.22—1.51 |

ALL = acute lymphoblastic leukemia; AML = acute myelogenous leukemia; BMI = body mass index; CI = confidence interval; CML = chronic myelogenous leukemia; CHF = chronic heart failure; CKD = chronic kidney disease; HCT-CI = Hematopoietic Cell Transplantation (HCT)-specific Comorbidity Index; IBD = inflammatory bowel disease; IQR = interquartile range; MDS = myelodysplastic syndrome; NHL = Non-Hodgkin lymphoma; SD = standard deviation; WBC = white blood cell; BUN = blood urea nitrogen.

**Supplemental Table 3: Associations between patient characteristics and primary CDI using random forest modeling in the derivation cohort.** Higher mean decrease Gini values denote predictors which contributed more to decision splits that meaningfully reduce Gini impurity. C-statistic = 0.70 in the internal validation cohort

| Characteristic | Mean Decrease Gini |
| --- | --- |
| Age | 27.73 |
| Max WBC | 25.79 |
| WBC | 25.18 |
| BMI | 25.25 |
| Max Creatinine | 23.59 |
| Glucose | 21.90 |
| AST | 21.39 |
| Total Hospital Visits | 21.03 |
| BUN | 19.11 |
| Albumin | 18.19 |
| Bilirubin | 15.33 |
| HCT-CI | 13.23 |
| Hematological malignancy | 12.43 |
| Previous chemotherapy | 10.63 |
| Graft type | 7.42 |
| Stem cell source | 5.88 |
| Penicillins | 4.29 |
| Sulfonamides | 4.10 |
| Cephalosporins | 4.10 |
| Sex | 3.88 |
| Quinolones | 3.49 |
| Antidiarrheals | 3.30 |
| Macrolides | 2.28 |
| Clindamycin | 1.76 |
| Tetracyclines | 1.25 |
| Immunosuppression | 0.94 |
| Acid suppression | 0.34 |

**Supplemental Table 4: Associations between patient characteristics and recurrent CDI using multivariable logistic regression in the derivation cohort.** C-statistic = 0.61 in the internal validation cohort.

| Characteristic | Adjusted OR | 95% CI | P-value |
| --- | --- | --- | --- |
| Tetracyclines | 12.80 | 0.94—174.2 | 0.055 |
| TPN | 18.4 | 1.04—325.5 | 0.047 |
| Age | 1.04 | 0.98—1.11 | 0.17 |
| WBC | 1.17 | 1.07—1.29 | 0.001 |

**Supplemental Table 5: Associations between patient characteristics and recurrent CDI using random forest modeling in the derivation cohort.** Higher mean decrease Gini values denote predictors which contributed more to decision splits that meaningfully reduce Gini impurity. C-statistic = 0.56 in the internal validation cohort.

| Characteristic | Mean Decrease Gini |
| --- | --- |
| WBC | 1.90 |
| Hematological Malignancy | 1.38 |
| Glucose | 1.35 |
| Bilirubin | 1.29 |
| AST | 1.24 |
| Age | 1.12 |
| BMI | 1.10 |
| Max WBC | 1.07 |
| Max Creatinine | 0.89 |
| Total hospital visits | 0.76 |
| BUN | 0.66 |
| HCT-CI | 0.44 |
| Albumin | 0.42 |
| Previous chemotherapy | 0.29 |
| Macrolides | 0.26 |
| Insurance | 0.24 |
| Opioids | 0.23 |
| Sex | 0.19 |
| Acid suppression | 0.15 |
| Antidiarrheals | 0.14 |
| Stem cell source | 0.12 |
| Quinolones | 0.11 |
| Sulfonamides | 0.10 |
| Penicillins | 0.09 |
| Graft type | 0.07 |
| Clindamycin | 0.07 |
| Cephalosporins | 0.06 |
| Tetracyclines | 0.06 |
